# Ophthalmic Simulated Surgical Competency Assessment Rubric for manual small-incision cataract surgery

**DOI:** 10.1016/j.jcrs.2019.04.010

**Published:** 2019-09

**Authors:** William H. Dean, Neil L. Murray, John C. Buchan, Karl Golnik, Min J. Kim, Matthew J. Burton

**Affiliations:** 1 International Centre for Eye Health, London School of Hygiene and Topical Medicine, London, England; 2 Imperial College London, London, England; 3 Tropical Epidemiology Group, Faculty of Infectious Disease Epidemiology, London School of Hygiene and Tropical Medicine, London, England; 4 Moorfields Eye Hospital, London, England; 5 Royal Australian and New Zealand College of Ophthalmologists, Sydney, Australia; 6 International Council of Ophthalmology, San Francisco, California, USA

## Abstract

**Purpose:**

To develop and test the validity of a surgical competency assessment tool for simulated small-incision cataract surgery (SICS).

**Setting:**

Participating ophthalmologists contributed from 8 countries.

**Design:**

Qualitative and quantitative development and evaluation of face and content validity of an assessment rubric, and evaluation of construct validity and reliability.

**Methods:**

The SICS Ophthalmic Simulated Surgical Competency Assessment Rubric (Sim-OSSCAR) was developed and assessed for face and content validity by an international group of experienced ophthalmologists. Groups of novice and competent surgeons from 4 countries were recorded performing surgery, and masked assessments were performed by 4 expert surgeons, to determine construct validity and reliability.

**Results:**

The Sim-OSSCAR for SICS was assessed by a panel of 12 international experts from 8 countries. In response to the question, “Do you think the OSSCAR represents the surgical techniques and skills upon which trainees should be assessed?,” all respondents either agreed or strongly agreed. Face validity was rated as 4.60 (out of 5.0). The content was iteratively agreed to by the panel of experts; final content validity was rated as 4.5. Interobserver reliability was assessed, and 17 of 20 items in the assessment matrix had a Krippendorff α correlation of more than 0.6. A Wilcoxon rank-sum test showed that competent surgeons perform better than novices (*P* = .02).

**Conclusions:**

This newly developed and validated assessment tool for simulation SICS, based on the International Council of Ophthalmology's Ophthalmology Surgical Competency Assessment Rubric, has good face and content validity. It can play a role in ophthalmic surgical education.

Cataract is the most common cause of blindness, accounting for 12.6 million of the 36-million blind people worldwide, along with 52.6-million people with moderate or severe vision impairment.^1^ Small-incision cataract surgery (SICS) is a widely accepted, appropriate, and affordable procedure that can deliver high-quality visual outcomes.^2^, ^3^, ^4^, ^5^

SICS is one of the most commonly performed surgical procedures worldwide.^6^, ^7^ Therefore, training ophthalmologists to perform the operation safely and efficiently is of major ophthalmic public health significance. Despite this need, concerns remain in several regions over the safety, quality, and efficiency of surgical training for cataract surgery.^6^, ^8^ The use of simulation-based surgical education, before and during the initial period of “live” surgery training, potentially has much to contribute. There is, however, a paucity of data on efficacy of simulation-based surgical education for the SICS technique. Therefore, as a first step to address this evidence gap, we have designed a surgical-skill assessment tool for use during simulation-based training, based on the International Council of Ophthalmology's Ophthalmology Surgical Competency Assessment Rubric (ICO-OSCAR).^9^

Surgical education is a journey characterized by gradually increasing knowledge and skill. Surgeons begin their training as “novices,” and with time spent observing and learning, they progress to being an “advanced beginner.” Someone who is “competent” can perform a task independently to a standard that is acceptable, though it might lack refinement.^10^ Surgeons who are “proficient” have developed a deep understanding and are able to see actions and situations more holistically. “Expert” surgeons can cope with and adapt to complex and new situations. This is the Dreyfus model of skills acquisition and expertise.

The Ophthalmic Simulated Surgical Competency Assessment Rubric (Sim-OSSCAR) was developed to aim toward the stage of “competence.” Using the Sim-OSSCAR as a learning and formative assessment tool, with a simulation eye, the novice SICS trainee would become competent. It is envisaged that a trainee should proceed to supervised surgery training on patients in the operating theater only after having attained the competence stage.

In the domain of medical and surgical education, validity refers to the degree to which an instrument measures what it sets out to measure. Content validity is whether the test measures a specific skill, and not other aspects such as anatomical knowledge. Face validity describes whether the chosen tasks resemble those that are performed during a surgical procedure in a real-life situation. Inter-rater reliability is the degree of agreement amongst different graders, and it will provide a measure of consensus.

The aim of the current study was to develop and validate a tool for use within training programs to assess trainee surgeons performing SICS. The ICO-OSCAR template was selected as the starting point and redesigned for assessing a simulated SICS surgical technique on an artificial eye. This Sim-OSSCAR was then deployed in conjunction with the use of an artificial eye specifically developed for SICS.^A^

## Materials and methods

### Sim-OSSCAR Content Revision and Development

The ICO OSCAR for SICS was developed by experts at the ICO using a modified Dreyfus scale (novice, beginner, advanced beginner, and competent).^11^, B The “proficient” and “expert” steps of the scale were excluded. In this study, the original ICO-OSCAR was modified to develop an assessment and training tool for simulated ophthalmic surgical education in SICS surgery. The ICO-OSCAR was initially edited to remove content not appropriate for simulation-based surgical training. The OSCAR was further adapted to a modified three-stage Dreyfus scale (novice, advanced beginner, competent). The draft of the Sim-OSSCAR was sent to a panel of 8 international content experts for further amendments to the content and structure of the Sim-OSSCAR. These people were selected for their experience and expertise in performing and teaching SICS. Responses were collated and synthesized into a final version of the rubric, which was distributed for further review.

### Face and Content Validity Assessment

Face and content validity were assessed using a standardized closed question evaluation on a 5-point Likert scale. This was done by a group of 12 international expert SICS cataract surgeons remotely via email, half of whom had been involved in the initial revision process. These SICS surgeons were selected based on their expertise and to ensure international representation. They teach and perform SICS surgery in Angola, Argentina, Ghana, Haiti, India, Malawi, Nepal, New Zealand, United Kingdom, and the United States. Surgeons were asked, “Do you think the Sim-OSSCAR represents the surgical techniques and skills upon which trainees should be assessed?” and “Would you change any of the cells/content? (If so, please include specific details).” Surgeons were also asked, “Do you think the Sim-OSSCAR (used with the artificial eye) is an appropriate way to assess trainees' surgical skill?” Responses on the 5-point Likert scale were given a numerical value and entered onto an Excel spreadsheet (Microsoft Corp.) before calculating the means ± SD. After the initial face and content validation round, three further minor amendments were made to the Sim-OSSCAR, and this validation process was repeated.

### Interobserver Reliability Assessment

To assess interobserver Sim-OSSCAR grading reliability, 8 simulated SICS procedures, which were performed by 8 separate cataract surgeons, were recorded. Four of the surgeons were novice trainee surgeons and 4 were experienced ophthalmologists (who had performed more than 100 SICS procedures). The procedures were performed on the SICS-specific artificial eye, made by Phillips Studio, and recorded using a Stemi 305 microscope with AxioCam ERc5s camera and Labscope digital classroom (all Carl Zeiss Meditec AG). The videos were anonymized so that the people doing the scoring were masked to the level of the trainee. The recordings were independently graded by 4 expert SICS surgeons who currently or had previously worked in high-volume training ophthalmology units in Ethiopia, India, Malawi, the Western Pacific region, and Sierra Leone. Each surgeon independently scored the videos of 8 simulation SICS procedures using the Sim-OSSCAR.

### Analysis

Data were managed in Excel and analyzed with Stata software (version 15.1, StataCorp, LLC). Krippendorff α was selected as the inter-rater agreement coefficient because there were multiple raters providing nonbinary ordinal scores. This was calculated separately for each of the 20 steps of the Sim-OSSCAR on a three-point ordinal point scale (0, 1, or 2). A value of 0.60 was deemed acceptable for a newly developed rubric.^12^, ^13^ A Wilcoxon rank-sum test was performed using the ranks for mean scores for novice and competent surgeons.

The validation study was approved by the Medicine Education Ethics Committee, Faculty Education Office (Medicine), Imperial College, London (MEEC1415-12), and the London School of Hygiene & Tropical Medicine ethics committee (11795).

## Results

### Sim-OSSCAR Content Revision and Development

An international reference group of 8 surgeons from 6 countries contributed to the initial development of the SICS Sim-OSSCAR. Table 1 shows the changes that arose from the editing of the ICO-OSCAR. The steps of draping, cauterization, irrigation/aspiration, and iris protection were removed. This group provided feedback on the content of the SICS Sim-OSSCAR. The discussion focused on anesthesia; preparation of the ocular surface; sterilizing the surgical field with povidone–iodine; conjunctival incision with flap, cautery, or hemostasis; decreasing pupil size; iris prolapse; and irrigation/aspiration clearance of cortical lens material. Comments regarding the global indices content also included adequacy of anesthesia and preparation. Consensus was reached that these content suggestions (Table 1) could be excluded from the Sim-OSSCAR because they largely related to live surgery and could not be simulated either by the artificial eyes or animal eye models. The initial Sim-OSSCAR was approved by the panel.

**Table 1:** Initial editing of ICO-OSCAR for small-incision cataract surgery to develop the Sim-OSSCAR.

ICO-OSCAR Item Label	Action/Change	Sim-OSSCAR Item Label
Draping	Removed	
Scleral access and cauterization	Removed cauterization and edited	Scleral incision
Irrigation/aspiration technique with adequate removal of cortex	Removed	
Wound closure (including suturing, hydration, and checking security as required)	Edited – suturing and hydration of wound removed	Procedure finish
Conjunctival and corneal tissue handling	Edited – reference to conjunctival tissue removed	Scleral and corneal tissue handling
Iris protection	Removed	
Overall speed and fluidity of procedure	Edited – Fluidity included as separate item, times adjusted	Overall speed of procedure

ICO-OSCAR = International Council of Ophthalmology's Ophthalmology Surgical Competency Assessment Rubric; Sim-OSSCAR = Ophthalmic Simulated Surgical Competency Assessment Rubric

### Face and Content Validity

The face and content validity were independently assessed by a group of 12 surgeons (6 of whom were in the initial reference group of 8). In response to the Face Validity question, “Do you think the Sim-OSSCAR (used with the artificial eye) is an appropriate way to assess trainees' surgical skill?,” all 12 of the respondents either agreed or strongly agreed. Overall, face validity was rated as 4.60 ± 0.52 out of 5 as a mean summation of 12 separate scores.

In response to the Content Validity question, “Do you think the Sim-OSSCAR represents the surgical techniques and skills upon which trainees should be assessed?,” all 12 respondents either agreed or strongly agreed. The content was finally agreed upon by the panel of experts, and the content validity was rated as 4.5 (out of 5).

### Interobserver Reliability

Interobserver reliability was assessed by an international panel of 4 experts in SICS. Eight separate masked video recordings of simulation SICS were sent to each expert surgeon for scoring using the Sim-OSSCAR. The recorded procedures represented a range of surgeon skills from complete novice to competent. The mean score for “novices” was 1.7 ± 1.0, and the mean score for “competent” SICS surgeons was 31.0 ± 2.7, out of a maximum score of 40.

To assess the interobserver agreement on the specific items in the Sim-OSSCAR, Krippendorff α coefficients were calculated. Table 2 shows the results for all 20 items in the Sim-OSSCAR, of which 17 exhibited an inter-rater agreement coefficient of Krippendorff α greater than 0.60. Three items had a lower Krippendorff α coefficient: “capsulotomy/capsulorhexis start,” “eye positioned centrally,” and “overall fluidity of the procedure.”

**Table 2:** Inter-rater Krippendorff α correlation for 20 facets of the Sim-OSSCAR.

Facet	Item	Krippendorff α	Percent Agreement
Specific step			
1	Scleral fixation	0.660	0.792
2	Paracentesis	0.663	0.792
3	OVD insertion	0.773	0.854
4	Scleral incision	0.869	0.917
5	Scleral tunnel	0.900	0.938
6	Sclerocorneal tunnel	0.896	0.938
7	Corneal entry	0.617	0.750
8	Capsulotomy/capsulorhexis start	0.414	0.604
9	Capsulotomy/capsulorhexis completion	0.767	0.854
10	Hydrodissection	0.782	0.875
11	OVD insertion	0.685	0.813
12	Prolapse of nucleus partially into AC	0.677	0.792
13	Nucleus extraction	0.894	0.938
14	IOL insertion	0.673	0.792
Global indices			
15	Corneal distortion	0.894	0.938
16	Eye positioned centrally within microscope view	0.394	0.583
17	Scleral and corneal tissue handling	0.880	0.938
18	Intraocular spatial awareness	0.796	0.875
19	Overall fluidity of procedure	0.518	0.708
20	Overall speed of procedure	1.000	1.000

AC = anterior chamber; IOL = intraocular lens; OVD = ophthalmic viscosurgical device; Sim-OSSCAR = Ophthalmic Simulated Surgical Competency Assessment Rubric

### Construct Validity

Construct validity is an assessment of the “sharpness” of a tool: can it discriminate between two distinct groups? For this study, these groups are the novice and competent surgeons. Table 3 shows the total score for each separate grader for all 8 videos. Novice surgeons were graded with a mean score range of 0.50 to 3.25 (out of 40), with standard deviations varying between graders' scores of 0.50 to 2.06. Competent surgeons were graded with a mean score range of 21.5 to 36.5 (with standard deviations varying from 0.58 to 4.51). A Wilcoxon rank-sum test showed that competent surgeons perform better than novices (*P* = .02).

**Table 3:** Total score correlation.

Video	Grader Score: n/40				Mean ± SD
	A	B	C	D	
1	5	1	2	5	3.25 ± 2.06
2	2	2	1	2	1.75 ± 0.50
3	2	2	1	0	1.25 ± 0.96
4	0	0	1	1	0.50 ± 0.58
5	39	37	33	37	36.5 ± 2.52
6	25	24	15	22	21.5 ± 4.51
7	36	35	29	34	33.5 ± 3.11
8	33	33	32	32	32.5 ± 0.58

## Discussion

Globally, 65.2-million people are blind or moderate/severely vision impaired because of cataract.^1^ Twenty-eight percent of countries have less than 4 ophthalmologists per one-million people.^14^ By subregion, the lowest mean ratio is 2.7 ophthalmologists per one million in Sub-Saharan Africa. There is a disproportionately high prevalence rate of cataract blindness in regions with the fewest ophthalmologists and cataract surgeons. There is a huge need for an increased number of well-trained ophthalmic surgeons, both ophthalmologist and nonphysician cataract surgeons to tackle this burden. There is a growing appreciation of the role of simulation in surgical education, especially in the initial acquisition of competence.

**Figure 1:**
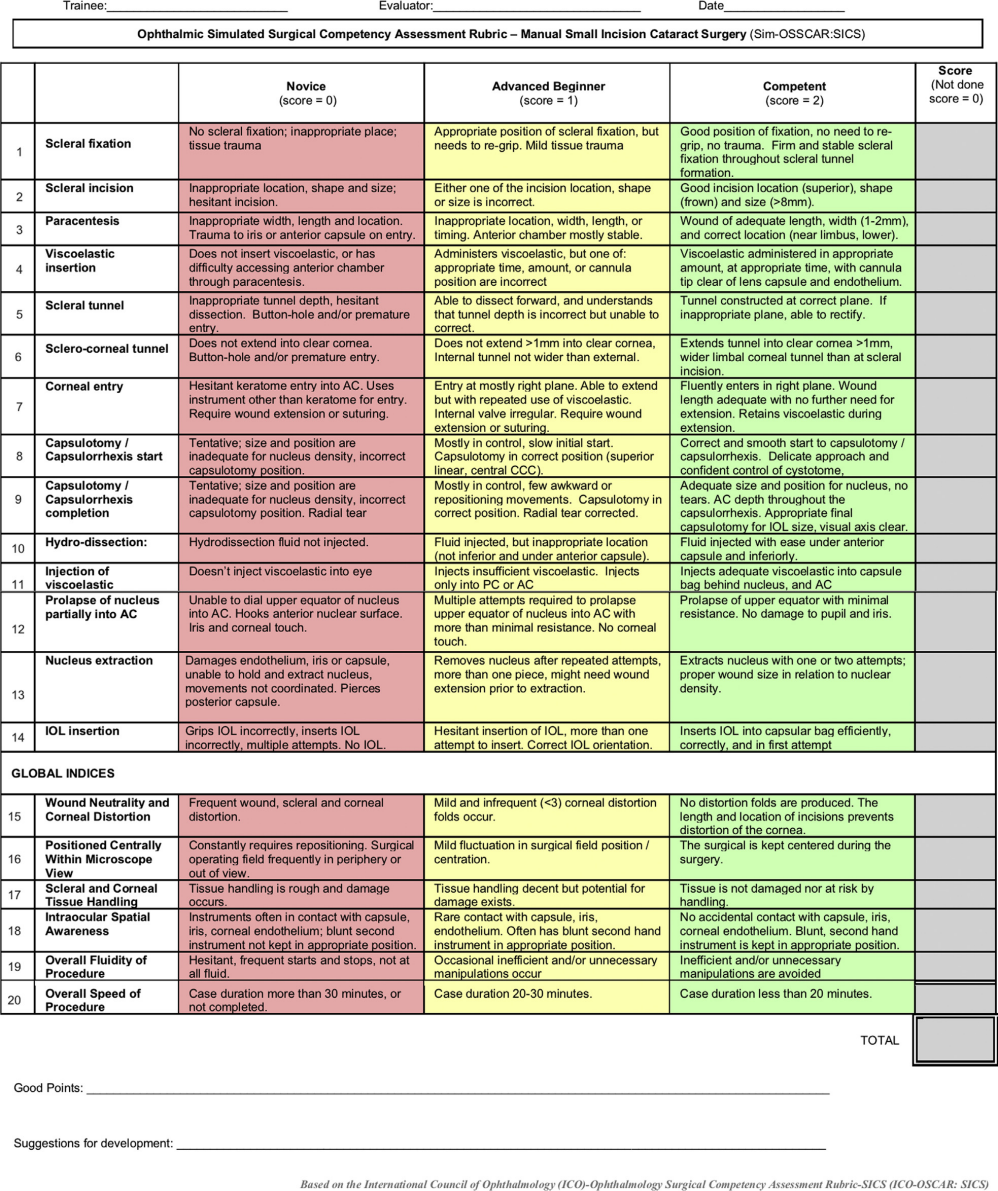
The Ophthalmic Simulated Surgical Competency Assessment Rubric for manual small-incision cataract surgery (Sim-OSSCAR:SICS).

The SICS Sim-OSSCAR (Figure 1) was developed to provide a formative assessment tool for initial cataract surgical training. The Sim-OSSCAR for SICS has good face and content validity as well as interobserver reliability and construct validity. It is important to note that face and content validity were quantified using closed-ended questions. Although open-ended comments were invited, we accept that this is a potential source of response bias.

Fidelity is important in simulation-based surgical education. Animal eyes have been used for training; however, the tissue feel in terms of rigidity or elasticity is different than human eyes. Animal eyes have a small window of fidelity before they disintegrate, cannot be used as a “standardized” training model, and often need preparation with formalin (aqueous solution of formaldehyde).^15^, ^16^ Artificial eyes offer standardization, and overall fidelity was rated as “high” or “very high” by 79% of the trainees on SICS courses (manuscript in preparation). Fidelity of scleral tunnel formation and capsulorhexis steps of SICS were rated “high” or “very high” by 100% of the trainees.

The OSACSS (Objective Structured Assessment of Cataract Surgical Skill) was developed as an objective performance-rating tool.^17^ The grading system contained global as well as phacoemulsification cataract surgery task-specific elements. Significant improvements in live surgical procedures have been shown after virtual reality cataract surgery training, as assessed by OSACSS.^18^ The OASIS (Objective Assessment of Skills in Intraocular Surgery) was also developed for phacoemulsification cataract surgery as an objective ophthalmic surgical evaluation protocol to assess surgical competency.^19^ The SPESA (Subjective Phacoemulsification Skills Assessment) assesses trainee performance in cataract surgery by combining a global approach, assessing detailed stage-specific criteria of critical components of cataract surgery.^20^

The ICO-OSCARs were originally based on the OSACSS; however, they were expanded upon by creating a set of behaviorally anchored scoring matrices that explicitly and precisely define what is expected for each step. The rubric was based on a modified Dreyfus model^10^; however, the final “expert” category was omitted because trainees were not expected to become experts during training. The ICO-OSCAR, as well as all other valuation tools described above, are aimed at assessment of surgical competence in the live operating theater setting. This currently validated Sim-OSSCAR is for use with SICS rather than phacoemulsification surgery, and it is aimed for use in a simulation surgical skill's center before live surgical training has commenced. It can be used during initial instruction, whereby the trainee SICS surgeon uses it as a clear list of the steps of the procedure. It can be used as a guide of what exactly is expected for each step to be deemed “competent.”

Although models have been available for modern phacoemulsification cataract surgery for over a decade, no artificial eyes had been previously developed for SICS. A full-immersion computerized SICS simulator is in the final stages of development; however, it is not yet widely available.^21^

The primary aim of the SICS Sim-OSSCAR is to provide a formative assessment tool. It could be used as a summative assessment tool upon which to progress the successful trainee to live supervised surgical training in SICS. It may be left to the trainer or training institution to benchmark appropriately, depending on the setting and educational goals. An example might be to require a mean of 75% score (30/40) over three cases, and no “zero” scores in any of the 20 steps.

Kappa measures (such as Krippendorff α) correct for chance agreement as the coefficients tend to punish variables with strongly skewed distributions. This explains the higher percentage agreements in Table 2. Three steps of the SICS Sim-OSSCAR had a lower interobserver reliability, with a Krippendorff α less than 0.60. These three steps were the starting of the capsulotomy, centration, and fluidity.

First, separate techniques for starting a capsulotomy or capsulorhexis exist in conventional cataract surgery: a continuous curvilinear capsulorhexis, linear (or envelope) capsulotomy, and a can-opener technique. Different cataract surgeons will themselves have subtle variations within these. Second, a limitation of the Stemi 305 microscope and Labscope App is the high zoom when recording, relative to what the surgeon sees through the binocular eyepieces. Finally, “fluidity” is by definition a subjective term and description.

We hope that the use of the newly developed Sim-OSSCAR will assist eye surgeon trainees in gaining competence and confidence within simulation-based surgical education, before then progressing to supervised live surgery.

We present a newly validated learning and assessment tool for simulation-based surgical education in cataract surgery. Its aim is ultimately to guide and assess initial simulation surgical training in SICS, to then give trainees the green lights to progress to live supervised surgery.

**What Was Known**
• Ophthalmology surgical competency assessment tools exist for live cataract surgical evaluation.

**What This Paper Adds**
• Surgical competency can be reliably measured for simulated cataract surgery.
